# A Randomized, Double-Blind, Placebo-Controlled Study of an Anthocyanin-Rich Functional Ingredient on Cognitive Function and Eye Dryness in Late Adulthood Volunteers: Roles of Epigenetic and Gut Microbiome Modulations

**DOI:** 10.3390/nu15163499

**Published:** 2023-08-08

**Authors:** Jintanaporn Wattanathorn, Terdthai Tong-un, Wipawee Thukham-mee, Pongsatorn Paholpak, Poonsri Rangseekhajee

**Affiliations:** 1 Department of Physiology, Faculty of Medicine, Khon Kaen University, Khon Kaen 40002, Thailand; terdthai@kku.ac.th (T.T.-u.); meewep@gmail.com (W.T.-m.); 2 Research Institute for High Human Performance and Health Promotion, Khon Kaen University, Khon Kaen 40002, Thailand; 3 Department Psychiatry, Faculty of Medicine, Khon Kaen University, Khon Kaen 40002, Thailand; ppaholpak@kku.ac.th (P.P.); poonsri@kku.ac.th (P.R.)

**Keywords:** anthocyanins, cognitive function, eye dryness

## Abstract

Due to the rising demand for supplements targeting cognitive enhancement and dry eye together with the health benefits of anthocyanins, we have developed a functional soup containing an anthocyanin-rich functional ingredient, or “Anthaplex,” and assessed the effects on cognitive function and eye dryness together with the possible mechanisms. A total of 69 male and female health volunteers were randomized and divided into placebo, D2, and D4 groups. All subjects consumed 120 mL of placebo or functional soup containing “Anthaplex” either at 2 or 4 g per serving per day within 5 min in the morning for eight weeks. The cognitive function, working memory, dry eye, AChE, MAO, MAO-A, MAO-B, and GABA-T activities, BDNF, HAC, HDAC, and DNMT activities, pH, and amount of lactic acid-producing bacteria, particularly *Lactobacillus* and *Bifidobacterium* spp. in feces, were determined before intervention and after eight weeks of consumption. Subjects who consumed the “Anthaplex” soup had improved cognitive function, working memory, eye dryness, histone acetylation, ACh E suppression, and BDNF with increased *Bifidobacterium* spp. but decreased pH in feces. These data suggest that “Anthaplex” improves cognitive function and eye dryness via the modulations of the histone acetylation process, gut microbiome, and cholinergic function.

## 1. Introduction

The global market of functional foods targeting wellness promotion, disease risk reduction, and quality of life promotion has been increasing around 8% [1,2]. Due to this demand, the development of novel functional ingredients is essential when producing new fortified foods and beverages. One of the functional ingredients that has gained much attention is herbal extracts due to the belief that natural products can provide better advantages with less side effects than synthetic compounds [3]. According to traditional medicine, both the whole plants and the mixture of plants are preferred more than the single pure compound owing to the synergistic interaction among various ingredients [4,5]. Therefore, the possibility of using a mixture of crude herbal extracts as a functional ingredient has been raised.

It has been revealed that anthocyanin-rich substances improve cognitive function by improving the dynamic balance of neurotransmitters, oxidative stress, inflammation, apoptosis, and signal transduction [6,7,8,9,10,11,12,13]. In addition, anthocyanin-rich substances also improve vision and eye health by improving oxidative stress status [14,15,16]. In addition to anthocyanin-rich substances, accumulative evidence during this decade has revealed that dietary fiber deprivation induces cognitive impairment by modulating the gut–brain axis [17]. Moreover, dietary fiber intake has also been shown to have a positive association with cognitive function in older adults [18]. Dietary fiber intake, particularly soluble fiber, decreases the risk of dementia [19]. Dietary fiber improves not only cognition but also eye health [20,21]. It has been demonstrated that dietary fiber modulates the gut microbiome giving rise to the improvement of cognition, and eye dryness [17,22,23,24,25,26,27,28]. Owing to the beneficial effects of anthocyanins and dietary fiber on cognition and eye health, together with the synergistic effect according to traditional medicine concepts, we hypothesized that a novel functional ingredient containing a mixture of an anthocyanin-rich substance and dietary fiber may possibly improve cognition and eye health, particularly eye dryness.

“Anthaplex” is a novel functional ingredient consisting of the extracts of purple waxy corn (*Zea mays*) and colored sticky rice (*Oryza sativa*). It is rich in anthocyanins and dietary fiber. It reveals antioxidant activity and can suppress acetylcholinesterase (AChE), monoamine oxidase (MAO), and GABA-transaminase (GABA-T). Therefore, “Anthaplex” may be a promising potential functional ingredient to improve age disorders such as cognition, and eye health, particularly eye dryness. Since no clinical evidence regarding this issue has been available until now, we aimed to determine the effect of the eight-week consumption of a functional soup containing “Anthaplex” on cognitive function and eye dryness in healthy volunteers in late adulthood. The possible underlying mechanisms were also explored.

## 2. Materials and Methods

### 2.1. Preparation of the Functional Soup

Purple waxy corn seeds (*Zea Mays* L; KKU open pollinated) were harvested, authenticated, and kindly provided by Associate Professor Dr. Bhalang Suriharn, Faculty of Agriculture, Khon Kaen University, Khon Kaen, Thailand. They were immersed in distilled water at a ratio of 1:2 (*w*/*v*) to provide a moisture content of around 30–31%. After soaking, water was poured out, and the corn seeds were washed again. Then, they were put in a plastic box and placed in an incubator at 35 ± 2 °C for 48 h or until the root germination was around 1 cm or more. The germinated corn was then dried at 50 ± 1 °C, baked until the moisture content was about 10 ± 2%, and then ground into a powder. Following this step, the ground powder was filtered through a 60-mesh sieve. Then, it was mixed with JWF 1/2564 ingredient (trade secret), which contained total anthocyanin content of around 1.608 ± 0.653 mg C3Gg of extract (dry weight) at a ratio of 1:3 to form “Anthaplex”, a novel functional ingredient.

**Figure 1:**
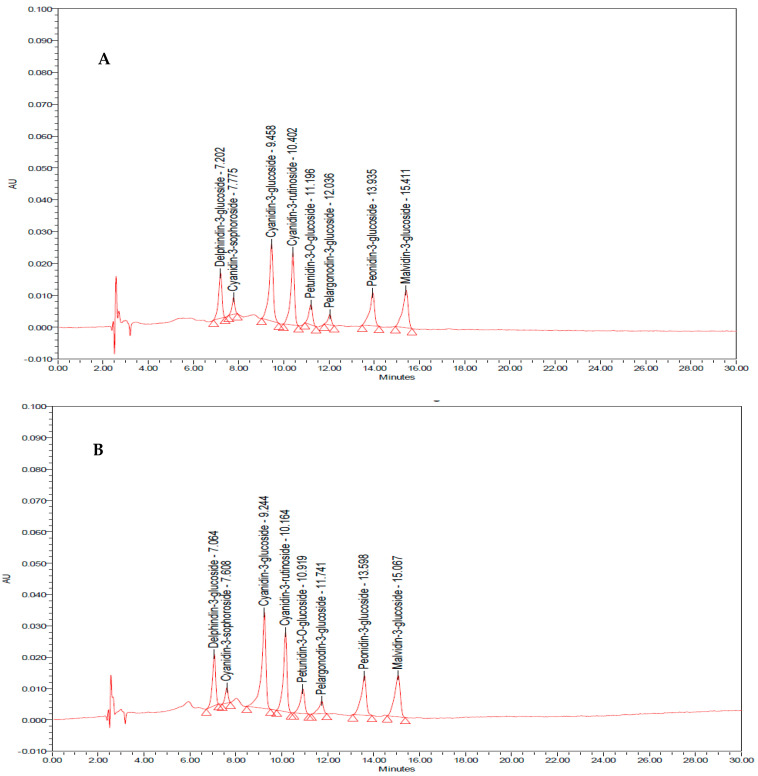
The fingerprint chromatogram of the anthocyanins in the functional soup containing “Anthaplex” at the doses of 2 g (**A**) and 4 g (**B**) per serving.

To prepare the functional soup, “Anthaplex” was mixed with a dairy-based soup containing a rice mixture (white color and rice berry, soy, pumpkin, onion, beetroot, parsley, milk, brown sugar, salt, rice bran oil, and water (under petty patent registration)) at doses of 2 and 4 g. The functional soup was assessed for polyphenolic compounds, flavonoids, and anthocyanin contents. In addition, chemical analysis of the instant functional soup was performed to determine the carbohydrate, protein, fat, energy, dietary fiber, and sodium contents by the Central Laboratory (Thailand) Co, Ltd., Khon Kaen, Thailand, a standard organization playing a role in inspection and quality and product certification. Various forms of anthocyanins were also assessed by using high-performance liquid chromatography (HPLC) [29,30,31,32,33]. The contents of delphinidin-3-glucoside, cyanidin-3-sophoroside, cyanidin-3-glucoside, cyanidin-3-rutinoside, petunidin-3-O-beta-D-glucoside, pelargondin-3-glucoside, and malvidin-3-glucoside in functional soup containing “Anthaplex” at the doses of 2 g and 4 g/serving are shown in Table 1 and Figure 1. In addition, it also contained dietary fiber content of around 1.68 and 1.89 mg per serving of functional soup at doses of 2 g and 4 g per serving.

### 2.2. Study Design and Population

An 8-week, 3-arm, randomized, double-blind, placebo-controlled study was performed to determine the memory and dry eye improvement effects. This study was approved by Center for Ethics in Human Research, Khon Kaen University, Khon Kaen Province, Thailand (HE641120). The protocol was also registered with Thai Clinical Trials Registry (TCTR 20210316008).

#### 2.2.1. Sample Size

The calculation of sample size was performed using 80% power and 95% level of confidence. This calculation was based on similar study, which was carried out in healthy volunteers [34], and N was 20/group. We also estimated withdrawal cases at around 10%, and this gave rise to 23/group.

#### 2.2.2. Participants

A total of 76 male and female participants were recruited from the area nearby Srinagarind Hospital, Faculty of Medicine, Khon Kaen University, Thailand. The inclusion criteria were described as follows: (1) aged between 45 and 65 years old, (2) 18 < BMI < 25 kg/m^2^, (3) adequate understanding of verbal and written Thai language, (4) no motor disability, no impairments of vision or hearing, and (5) normal cognitive ability or Montreal Cognitive Score (MoCA score) ≥ 25. The exclusion criteria included (1) cardiovascular disease, respiratory disease, neuropsychiatric disorders, head trauma, diabetes mellitus, liver disease, kidney disease, cancer, autoimmune, allergy, and hematological disorders, (2) exposure to pesticides within 1 week prior to the study, (3) hysterectomy or oophorectomy, (4) participating in other clinical trials, (5) consumption of drugs or supplements that exert influence on the function of nervous system, (6) smoking > 10 cigarettes/day, (7) drinking alcoholic beverages > 5 glasses/day 7 days prior the start of study, and (8) exercising > 3 times/week. After the physical examination, only 71 participants were eligible to participate in the study. All participants provided written informed consent prior to particpating in the study. During the study, some subjects withdrew from the study (one placebo case and one D2 case due to loss of communication). Finally, 69 subjects participated in the completed study.

#### 2.2.3. Study Design

**Figure 2:**
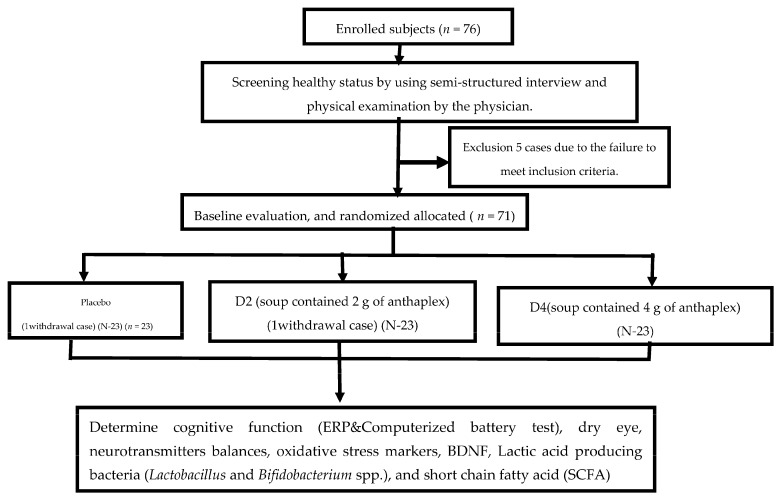
Schematic diagram of the experimental procedures.

A total of 69 participants were randomly assigned to placebo, D2, or D4 treatment groups. All assigned substances were matched for appearance, taste, volume, and calories (428 kcal). Each serving of the tested soups contained carbohydrates (72 g), fiber (10.5 g), fat (9.84 g), and protein (11.96 g). “Anthaplex” contains of a mixture of germinated purple corn and rice berry. No “Anthaplex” was provided to the placebo group, whereas experimental groups D2 and D4 were provided “Anthaplex” at dosages of 2 g and 4 g, respectively. However, purple corn was added to placebo and D2, respectively, to balance the total weight per serving. Information regarding characteristics, anthropometry, cognitive function, working memory, dry eye, serum activities of acetylcholinesterase (AChE), total monoamine oxidase (MAO), monoamine oxidase-A (MAO-A), monoamine oxidase-B (MAO-B), and 4-aminobutyrate aminotransferase (GABA-T) enzymes, and brain-derived nerve growth factor (BDNF) together with oxidative stress markers, including malondialdehyde (MDA) and the activities of superoxide dismutase (SOD), catalase (CAT), and glutathione peroxidase (GPx), was monitored at baseline and at the end of an 8-week study period. In addition, the amounts of lactic-acid-producing bacteria, *Lactobacillus* spp. and *Bifidobacterium* spp., short-chain fatty acids (SCFA) such as acetate, propionaye, and butyrate were also determined. The study protocol is summarized in Figure 2.

### 2.3. Outcome Measurement

#### 2.3.1. Cognitive Function Assessment

An assessment of cognitive function was widely performed by using a non-invasive event-related potential (ERP), an effective neurophysiological technique with low cost that is well suited for studying cognitive function of adolescents in physiological situations and in psychiatry [35]. It was used as a primary outcome. N100 brain wave, or the most negative peak occurring between 70 and 160 ms post-stimulus, has been reported to reflect the ability to allocate attention [36], whereas P300 component, or the most positive peak between 250 and 500 ms post-stimulus, reflects both attention and cognitive processing [37]. In this study, the brain wave was recorded via an international 10/20 lead system via a 40-channel electrode cap (Neuroscan, Inc., Sterling, FL, USA), and an auditory oddball paradigm was used to deliver the target and non-target tone bursts. The participants selected the response to either target or non-target tones by pressing the corresponding response button in front of them, as previously described [38]. The latency and amplitude of N100, P300 at Cz, and Fz locations were selected for the analysis because of their optimum changes of both peaks, as mentioned earlier in those recorded areas [35].

#### 2.3.2. Working Memory Assessment

Working memory was assessed by a battery set of memory tests consisting of word presentation, word recognition test, picture presentation, picture recognition test, simple reaction test, digit vigilance, choice reaction time, spatial, and numeric working memory, as previously mentioned [34]. Response time of simple reaction test, choice reaction time, and digit vigilance were assessed to reflect power of attention, whereas accuracy of digit vigilance and choice reaction time were assessed to reflect the continuity of attention. In addition, response times in word and picture recognition tests together with spatial and numeric working memory tests were monitored to assess the speed of memory, while an accuracy response in the tests just mentioned was used to reflect the quality of memory. Brief description of each test is provided below.

Word presentation test measured word recognition memory via recall capability. After a 15-word presentation (1 s/stimulus and 1 s for duration between stimuli), each subject had to recall the word that was previously presented as much as possible, and both response time and percentage of accuracy were recorded.

Word recognition test was applied to measure the recognition memory via recognition capability. In this test, each subject was exposed to a 30-word set, which consisted of 15 new words and 15 old words that were previously presented in the word presentation test. During the presentation, subjects pressed “yes” button when the word was previously presented and pressed “no” button when the word was a new one. The reaction time and percentage of accuracy were recorded.

Picture presentation test was performed in similar process as mentioned in the word presentation, except each subject was exposed to a set of 20 pictures, and each picture was presented for 3 s.

Picture recognition test was also a recognition test, as mentioned in the picture recognition test, but the subjects were subjected to a 40-picture set consisting of 20 new pictures and 20 pictures that were previously presented in the picture presentation.

Simple reaction time test measured the response time that each subject took to identify a stimulus on the monitor and press a response button.

Digit vigilance test was carried out to determine sustained attention capability and psychomotor speed. This test was designed to measure vigilance during rapid visual tracking. Each subject had to identify and match the presentation of each digit in the central part of monitor with the digit that was provided in the right corner and press the provided button as quickly as possible when the presentation was matched with the targeted digit. The response time and accuracy of responses were recorded.

Choice reaction time test was designed to measure alertness, or power of attention, and motor speed. Regarding this test, each subject was exposed to both targeted and non-targeted stimuli and had to press the provided button when the targeted stimuli were presented. Both response time and response accuracy were recorded.

Spatial working memory test measured ability to recall spatial information. Each subject was exposed to a picture of a house containing nine windows. Four windows were illuminated, and subjects had to memorize the location of them as targeted stimuli. During the test, a series of house pictures with one illuminated window were provided to each subject, and the subjects had to match the location of the presented picture with the location of the targeted stimuli and press the provided button as quickly as possible when the matching was correct. The speed and accuracy of responses were also monitored.

Numeric working memory test measured the ability to match 5 targeted numbers ranging from 0–9 that were presented on the screen before the test with a series of 30 numbers presented during the test. The subjects had to press the provided button as quickly as possible when the matching was correct, and both response time and accuracy of responses were recorded.

#### 2.3.3. Biochemical Assays

All blood samples were collected between 7:00 and 8:00 a.m. in a tube with clotting activator and left to coagulate at room temperature for 30 min. Then, all samples were prepared as serum by subjecting to 2000 g centrifugation at room temperature for 10 min. The serum supernatant was harvested and stored at −80 °C within 1 h of blood sampling and used for further investigations.

##### Oxidative Stress Marker Assessment

A measurement of serum oxidative stress markers, including malondialdehyde (MDA) levels and the activities of superoxide dismutase (SOD), catalase (CAT), and glutathione peroxidase (GPx), was determined by spectrophotometric method [39]. The plasmatic MDA level was quantified by thiobarbituric acid reactive substance assay. MDA value was calculated against the standard curve of 1,1,3,3-tetraethoxypropane. SOD activity was assessed by measuring the inhibition rate of cytochrome C reduction, whereas CAT was determined by monitoring the reduction in H_2_O_2_ absorbance, and GPx was assessed via the measurement of the reduction of reduced glutathione (GSH) generated by the reaction of hydrogen peroxide and 5,5′-dithiobis(2-nitrobenzoic acid) (DTNB, Ellman’s reagent).

##### Assessments of the Neurotransmitter Disturbances

The neurotransmitter disturbances, including the alterations of acetylcholine (ACh), gamma aminobutyric acid (GABA), and monoamine oxidase (MAO; total MAO, MAO-A, MAO-B)), were indirectly performed by measuring the suppression activities of acetylcholinesterase (AChE), GABA-transaminase (GABA-T), and all forms of monoamine oxidase (MAO) by using the modified method of Srichomphu and colleagues, as previously mentioned [40].

##### Assessment of Brain-Derived Neurotrophic Factor (BDNF), Histone Acetylase, Histone Deacetylase, and DNA Methyltransferase

Serum BDNF was assessed by using a commercial ELISA kit (ELISA; ab212166, Abcam, Cambridge, UK) with a high sensitivity that can detect less than 2.4 pg/mL BDNF. An assessment was performed according to the instructions provided by the manufacturer. All obtained values were calculated from the standard curve of BDNF at various concentrations [41]. Histone acetylase (HAT), histone deacetylase (HDAC), and DNA methyltransferase (DNMT) were assessed by using ELISA kit (ab 65352, ab156064, and ab 113467) according to the provided protocols of the company.

### 2.4. Assessment of Eye Dryness

Normal healthy eyes were screened by using the standard methods and procedures for opthalmic examination according to the guidelines of the World Health Organization (WHO) for assessing xeropthalmia and night blindness for physicians [42]. The severity of xeropthalmia was graded and changed to scale of 0–9, as described as follows. Normal eye = 0 (X0), night blindness (XN) = 1, conjunctival xerosis or dryness of conjunctiva (X1A) = 2, Bitot’s spot (X1B) or the presentation of white foamy lesion at bulbus conjunctiva = 3, corneal xerosis or dry cornea (X2) = 4, corneal ulceration or cornea showed ulceration (small punched-out in a corneal area <1/3 cornea) (X3A) = 5, corneal ulceration or keratomalacia (ulceration ≥2/3 cornea) = 6, corneal scaring (XS) = 7, xeropthalmic fundus (XF) = 8. Then, eye dryness was scored by using the modified method of Messmer [43]. The score was graded according to the following criteria: (1) discomfort, severity, and frequency of attack, (2) visual symptoms, (3) conjunctival injections, (4) corneal/tear signs, and (5) lids/meibomian glands. Each item was scored in the range of 1–4 as shown in Table 2. Then, the scores were together and gave rise to total score.

### 2.5. Determine of Enumeration of Lactobacillus spp. and Bifidobacterium spp.

A fecal sample of 1 g was diluted in 9 mL of phosphate buffer solution (pH 7.4), then diluted to 10^−6^–10^−7^ concentration. Samples at the volume of 100 μL from each initial concentration of sample were spread out onto a modified Man–Rogosa–Sharpe (MRS) media (Himedia, Maharashtra, India) (MRS agar) and HiCrome Bifidobacterium Agar (Himedia, India). The plates were then placed in an incubator at 37 °C for 48 h in anaerobic conditions by using a GasPak™ anaerobic (MGC, Mitsubishi, Tokyo, Japan). Each bacterial colony on MRS was counted as the total amount of lactic acid bacteria. Then, the number of *Lactobacillus* spp., and *Bifidobacterium* spp. were identified by using morphology and gram staining [44].

### 2.6. Short-Chain Fatty Acid (SCFA) Extraction and Determination

Short-chain fatty acids consisting of acetic, propionic, and butyric acids were extracted as the method previously described [45]. In brief, 20 g of feces was mixed with 200 μL of distilled water and homogenized. Then, the homogeneous sample was mixed with 200 μL of organic solvent containing N-butanol, tetrahydrofuran, and acetonitrile at a ratio of 50:30:20. Following this process, the mixture was mixed with 40 μL of 0.1 M HCl, 20 mg of citric acid and 40 mg of sodium chloride. After mixing, the mixture was vigorously shaken by using a vortex stirrer for 1 min. Then, the sample was subjected to 12,000 rpm centrifugation at room temperature for 10 min. At the end of the centrifugation process, the supernatant was harvested for SCFA assessment via gas chromatography/mass spectrometry (GC-MS) at Suranaree University of Technology. In brief, the supernatant at a volume of 100 μL was mixed with 10 μL of 5 M HCl, and pH was adjusted to 2. Then, a sample was extracted by adding 100 µL of anhydrous diethyl ether (DE) (1:1, *v*/*v*), vortexed, incubated on ice for 5 min, and subjected to a 10,000× *g* centrifugation for 5 min. At the end of centrifugation process, a DE layer that contained SFCA was transferred to a new microtube containing anhydrous Na_2_SO_4_ to remove water, and the extraction process was repeated 2 times. Then, 100 μL of DE extract was transferred into a glass insert in a GC vial, added to 5 µL of BSTFA, vortexed for 5 s, and capped tightly. The GC vial was subjected to incubation at 70 °C for 20–40 min and 37 °C for 2 h. After an incubation process, the derivatized samples were loaded into GC-MS. Pure water was processed with the same procedure and served as a blank sample. The background acetic acid was eliminated using a calculation from the peak area of sample minus the peak area of blank.

A sample separation was performed by using gas chromatography. SCFA separation was carried out by a Bruker 450 GC Rtx-5MS Capillary column (Bruker, Billerica, MA, USA) (30 m × 0.25 mm × 0.25 µm). The column temperature was held at a temperature of 45 °C for 2 min and raised to 250 °C at a rate of 4.5 °C/min. Thereafter, an aliquot of sample at a volume of 1 μL was injected at a split ratio of 100. Helium gas was used as carrier at a flow rate of 1 mL/min. Regarding mass spectroscopy, the temperatures of the electron source and the quadrupole were held at 230 °C and 150 °C, respectively. The energy of electron ionization was set at 70 eV. An analysis was performed in full scan mode from *m*/*z* range of 40–150. A compound identification was performed by comparing the retention time and MS spectra with those of the chemical standards. The detection was carried out by using the selected ion monitoring (SIM) mode. Data analysis was performed by using Wiley 9 GC-MS Library (Wiley, Hoboken, NJ, USA) [46].

### 2.7. Statistical Analysis

All experimental data were presented as mean ± standard error of the mean (S.E.M). All data were compared between placebo and experimental groups (D2 or D4), except severity score of eye dryness, which was also compared between baseline and after an 8-week consumption period. Statistical analysis was achieved by ANOVA followed by post-hoc Tukey’s test. Significant differences among groups were regarded when *p*-value < 0.05.

## 3. Results

### 3.1. Demographic Data of Subjects

Our data showed that no significant differences in the age, vital signs, height, body weight, and body mass index of subjects were observed before participating in this study, as shown in Table 3. However, after 8 weeks of consuming the functional soup containing “Anthaplex”, subjects who consumed the functional soup that contained “Anthaplex” at a dose of 4 g per serving showed a significant reduction in both systolic and diastolic blood pressure (*p*-values < 0.01 and 0.05, respectively, compared to the placebo group), as shown in Table 4.

### 3.2. Changes in Cognition

Table 5 revealed that no significant differences in latency and amplitudes of N100 and P300 among groups were observed at baseline. However, the brain wave at the Fz location of subjects who consumed the functional soup containing “Anthaplex” at a dose of 2 g/day significantly decreased N100 latency but increased N100 amplitude (*p*-value < 0.05 all; compared to placebo group). In addition, subjects who consumed the functional soup containing “Anthaplex” at a dose of 4 g per day showed a significant increase in N100 amplitude (*p*-value < 0.05; compared to placebo group). At the Cz location, a significant increase in the N100 amplitude was also observed in subjects who consumed the functional soup that contained “Anthaplex” at both doses mentioned earlier (*p*-value < 0.05 all; compared to the placebo group), as shown in Table 5.

In this study, we also measured working memory by using a computerized battery test, and the data are shown in Table 6. Prior to the consumption of the functional soup containing “Anthaplex”, no significant differences in both reaction time and percent of accuracy response among groups in the computerized battery test were observed. After 8 weeks of consumption, it was demonstrated that the reaction time of subjects who consumed the functional soup containing “Anthaplex” at a dose of 2 g per serving per day significantly decreased in the simple reaction time test (*p*-value < 0.05; compared to placebo). Furthermore, an increase in the percent of accuracy response in numeric working memory was also observed in subjects who consumed the functional soup containing “Anthaplex” at the doses of 2 g and 4 g per serving per day (*p*-value < 0.05 all; compared to the placebo). Therefore, the data suggested that the volunteers who consumed the functional soup containing “Anthaplex” significantly enhanced their power of attention and quality of memory.

### 3.3. Changes of Eye Dryness Severity

In this study, dry eye severity was examined according to the modified criteria of Messmer (2015) and the guidelines of the World Health Organization (WHO) for assessing xeropthalmia and night blindness for physician [43]. The data are shown in Table 7. No significant difference in the severity score of eye dryness was observed at baseline. After 8 weeks of consumption, subjects who consumed both doses of the functional soup showed a reduction in the severity score of eye dryness from baseline, while the placebo group failed to show the significant improvement. However, a significant difference was observed only in the group that received the functional soup at a dose of 2 g per day (*p*-value < 0.05).

### 3.4. Changes of Neurotransmitters and Brain-Derived Neurotrophic Factor (BDNF)

Prior to the consumption of the assigned substance, no significant differences in any parameters of the placebo and experimental groups were observed. After an 8-week consumption period, the subjects who consumed the functional soup at a dose of 2 g per day had significantly decreased serum AChE activity (*p*-value < 0.05; compared to the placebo). Furthermore, the significant elevation of serum BDNF was also observed in the subjects who consumed both doses of the functional soup at the end of the study period (*p*-value < 0.05 all; compared to the placebo), as shown in Table 8.

### 3.5. Changes in pH, Amount of Lactic Acid Producing Bacteria, Lactobacillus spp., and Bifidobacterium spp. in Feces

Our data showed that before the intervention, no significant differences in the feces pH, amount of lactic-acid-producing bacteria, *Lactobacillus* spp., and *Bifidobacterium* spp. in the feces of subjects in all treatment groups were observed. At the end of an 8-week study period, the feces pH of subjects who consumed the functional soup at both doses significantly decreased (*p*-value < 0.05 all; compared to the placebo). In addition, subjects who consumed the functional soup at a dose of 2 g per day also showed a significant increase in the amount of *Bifidobacterium* spp. (*p*-value < 0.01; compared to the placebo), as shown in Table 9.

### 3.6. Changes of Short-Chain Fatty Acid

The content of short-chain fatty acids consisting of acetate, propionate, and butyrate was assessed, and the data are shown in Table 10. After an 8-week consumption period, subjects in all intervention groups showed an increase in all types of short-chain fatty acids (SCFA). However, a significant increase of SCFA, particularly propionate, was observed only in subjects who consumed the functional soup containing “Anthaplex” at a dose of 4 g per day (*p*-value < 0.05; compared to the placebo group).

### 3.7. Changes in Epigenetic Mechanism via Histone Acetyltransferase (HAT), Histone Deacetylase (HDAC), and DNA Methyltransferase (DNMT)

Due to the crucial role of epigenetic modification on BDNF [47,48,49], we also determined the effect of the functional soup containing “Anthaplex” at the doses of 2 g and 4 g per serving per day. Table 11 showed that prior to the intervention, no significant changes in the mentioned parameters were detected among the groups. However, subjects who consumed functional soup at a dose of 4 g per day showed a significant elevation of histone acetylase, whereas subjects who consumed the functional soup at a dose of 2 g per day showed a significant reduction of histone deacetylase at the end of the study period (*p*-value < 0.01, and 0.05, respectively; compared to the placebo group). No significant change in DNMT was observed throughout the study period.

## 4. Discussion

The current data demonstrated that the functional ingredient “Anthaplex” in the developed functional soup increased the N100 amplitude in ERP, percent of accuracy response in numeric working memory, serum BDNF, and amount of *Bifidobacterium* spp. bacteria, propionate, and histone acetylase but decreased the N100 latency in ERP, severity score of eye dryness, AChE, feces pH, and histone deacetylase.

It has been reported that an increase in N100 amplitude reflects an increase in the synchronization of functional neurons during selective attention. This change gives rise to the improvement in the encoding process of information, whereas divided attention can produce a negative impact on working memory [50,51]. Thus, the aforementioned changes suggest that “Anthaplex” can improve the attention process, giving rise to an increase in encoding efficiency, resulting in the improvement of the cognition process. The current data also reveal that subjects who consumed the functional soup containing “Anthaplex” also improved their response times in the simple reaction time test, which confirms the improvement of their attention [52]. Furthermore, an increase in the percent of accuracy response in numeric working memory was recorded, which reflects an improvement in the quality of working memory [34,53,54,55].

It has been demonstrated that an increase in working memory is associated with the elevation of BDNF [55]. Accumulative evidence has also revealed that the expression of BDNF can be modified by epigenetic mechanisms such as the modification of histone [47,48,49]. The present data also demonstrated improved working memory and cognitive function together with changes in BDNF. In addition, our data also revealed that subjects in the study groups that were given “Anthaplex” showed improved cognitive function and working memory, together with the increased activity of BDNF and histone acetylase but decreased activity of histone deacetylase. Owing to the crucial role of histone modification on BDNF and working memory, as mentioned earlier together with the crucial role of AChE suppression on memory [55,55,56,57], we do suggest that the improvement of cognitive function observed in the subjects provided with “Anthaplex” may have occurred partly via the modification of the epigenetic mechanism by enhancing histone acetylase but suppressing histone deacetylase activities, giving rise to the elevation of BDNF and via the suppression of AChE.

BDNF exerts a crucial role not only in cognitive function but also in ocular health [58]. It has been reported that the reduction of BDNF can reduce tear secretion, giving rise to eye dryness [58,59]. However, our data show that despite the significant increase in BDNF in subjects provided with “Anthaplex” at a dose of 4 g per day, no significant improvement in the severity score of eye dryness was observed. This evidence suggests that other factors such as inflammation [60] and oxidative stress [61] may also play a role in the improvement of eye dryness. It has been reported that the regulation of the resting flow of tears is under the influence of the sensory impulse of the trigeminal nerve, which arises from the ocular surface and sends the fibers to the superior salivary nucleus in the pons, which sends efferent fibers in the nervus intermedius to the pterygopalatine ganglion, which in turn sends post-ganglionic fiber to lacrimal glands and vessels of orbit. Therefore, the disturbance of the ocular surface induced by either oxidative stress or inflammation or both factors can disturb the flow of tears, which in turn induces eye dryness [62]. In the current study assessment, the inflammatory and oxidative stress status of the eye surface was not measured but needs to be determined through further investigations.

A recent study has revealed that gut microbiota can also disturb the “gut dysbiosis–ocular surface–lacrimal gland axis”, which in turn induces a dry eye condition [63]. Therefore, we also determine the alteration of lactic-acid-producing bacteria, particularly *Lactobacillus* and *Bifidobacterium* spp. It has been shown that subjects who are treated with “Anthaplex” at a dose of 2 g per day showed increased amounts of *Bifidobacterium* spp. in feces. In addition, recent evidence clearly shows that *Bifidobacterium bifidum* can alleviate dry eye by decreasing inflammation [64]. Therefore, our data suggest that an improvement in eye dryness may occur partly via an improvement of BDNF induced by the modification of histone modification [38,47,58] and by the modification of gut microbiota, particularly *Bifidobacterium* spp. [65]. Furthermore, the modification of *Bifidobacterium* spp., which in turn enhances BDNF [66], also plays a role in the improvement of cognitive function and working memory, as observed in this study.

Accumulative lines of evidence have revealed that anthocyanins can modulate the epigenetic mechanism by enhancing histone acetylation [66,67]. Anthocyanins are also associated with an increase in the amount of *Bifidobacterium* spp. [68,69,70] and the suppression of AChE [55]. Owing to the mentioned information, we do suggest that the positive modulation effect induced by “Anthaplex” observed in this study may partially be associated with anthocyanins and their metabolites. However, further studies are required to confirm this proposal for identifying a precise understanding regarding this point. Furthermore, dietary fiber presented in the functional soup may also play a role [20,21].

Interestingly, “Anthaplex” at a dose of 4 g per serving per day also produced a significant reduction of both systolic and diastolic pressure. Although the mechanisms of this action are beyond the scope of this study, we suggest that “Anthaplex” at this dose may decrease arterial stiffness [71], which is in alignment with previous studies that show anti-arterial stiffness and antihypertension effects of anthocyanin [72]. However, this aspect still requires further research to confirm the mechanism.

## 5. Conclusions

The current data clearly demonstrate that “Anthaplex” can improve cognitive function and working memory together with eye dryness. The possible underlying mechanisms may occur via the modulations of the histone acetylation process and the gut microbiome, particularly in regard to *Bifidobacterium* spp. The suppression of AChE may also play a role in the improvement of cognition and memory. “Anthaplex” could therefore be an interesting new functional ingredient for supplements aiming to improve cognitive function and eye dryness.

## Figures and Tables

**Table 1:** The contents of various forms of anthocyanins in the functional soup containing “Anthaplex” at the doses of 2 g and 4 g/serving, respectively.

Substance	Concentration (mg/g Sample)	
	Soup Containing “Anthaplex” 2 g per Serving	Soup Containing “Anthaplex” 4 g per Serving
Delphinidin-3-glucoside	0.010 ± 0.002	0.011 ± 0.001
Cyanidin-3-sophoroside	0.0130 ± 0.001	0.016 ± 0.003
Cyanidin-3-glucoside	0.016 ± 0.004	0.021 ± 0.003
Cyanidin-3-rutinoside	0.016 ± 0.004	0.015 ± 0.001
Petunidin-3-O-beta-D-glucoside	0.013 ± 0.004	0.017 ± 0.004
Pelargondin-3-glucoside	0.014 ± 0.007	0.021 ± 0.003
Peonidin-3-O-glucoside	0.018 ± 0.002	0.022 ± 0.001
Malvidin-3-glucoside	0.015 ± 0.002	0.015 ± 0.001

**Table 2:** Dry eye disease severity grading scheme according to the modified method of Messmer.

Dry Eye Severity Level	1	2	3	4
Discomfort, severity, frequency	Mild and/or episodic; occurs under environmental stress	Moderate episodic or chronic stress or no stress	Severe frequent or constant without stress	Severe and/or disabling and constant
Visual symptoms	None or episodic mild stress	Annoying and/or activity-limiting episodic	Annoying and/or constant-limiting activity	Constant and/or possibly disabling
Conjunctival injection	None to mild	None to mild	+/−	+/+++
Corneal/tear signs	None to mild	Mild debris, ↓ miniscus	Filamentary keratitis, mucus clumping, ↓ tear debris	Filamentary keratitis, mucus clumping, ↓ tear debris, ulceration
Lid, meibomian glands	Meibomian gland dysfunction variably present	Meibomian gland dysfunction variably present	Frequent	Trichiasis, keratinization, symblepharon

+/−: irreversible change of conjunctiva without permanent visual loss, +/+++: irreversible change of conjunctiva with permanent visual loss. ↓: decrease.

**Table 3:** Demographic data of subjects before the intervention. Data were presented as mean ± SEM (*n* = 23/group).

Parameters	Baseline		
	Placebo	D2 g per Day	D4 g per Day
Age (year)	51.00 ± 0.85	50.30 ± 0.90 (*p* = 0.732)	51.13 ± 0.82 (*p* = 0.929)
Gender (Male/Female)	1/22	0/23	1/22
Body Temperature (°C)	36.25 ± 0.12	36.30 ± 0.08 (*p* = 0.748)	36.36 ± 0.08 (*p* = 0.738)
Heart Rate (beats/min)	72.39 ± 1.34	73.47 ± 1.95 (*p* = 0.361)	72.91 ± 1.36 (*p* = 0.668)
Respiratory Rate (breaths/min)	16.82 ± 0.20	16.73 ± 0.20 (*p* = 0.765)	16.47 ± 0.20 (*p* = 0.233)
Systolic Blood Pressure (mmHg)	119.05 ± 2.06	117.82 ± 1.64 (*p* = 0.613)	114.53 ± 1.39 (*p* = 0.078)
Diastolic Blood Pressure (mmHg)	78.56 ± 1.89	74.21 ± 1.66 (*p* = 0.091)	73.78 ± 1.81 (*p* = 0.064)
Body Weight (kg)	58.15 ± 1.76	59.17 ± 1.70 (*p* = 0.655)	58.48 ± 1.34 (*p* = 0.885)
Body Height (cm)	155.39 ± 0.99	155.39 ± 1.00 (*p* = 1.000)	155.47 ± 1.58 (*p* = 0.960)
Body Mass Index (BMI) (kg/m^2^)	24.02 ± 0.61	24.47 ± 0.61 (*p* = 0.618)	24.30 ± 0.67 (*p* = 0.757)

D2, D4: The function soup containing “Anthaplex” at the doses of 2 g and 4 g per serving.

**Table 4:** Demographic data of subjects after an 8-week consumption period of the functional soup containing “Anthaplex” at the doses of 2 g and 4 g per serving. Data were presented as mean ± SEM (*n* = 23/group).

Parameters	8-Week		
	Placebo	D2 g per Day	D4 g per Day
Age (year)	51.00 ± 0.85	50.30 ± 0.90 (*p* = 0.732)	51.13 ± 0.82 (*p* = 0.929)
Gender (Male/Female)	1/22	0/23	1/22
Body Temperature (°C)	36.43 ± 0.06	36.35 ± 0.07 (*p* = 0.547)	36.45 ± 0.06 (*p* = 0.982)
Heart Rate (beats/min)	74.30 ± 1.65	70.95 ± 1.66 (*p* = 0.158)	76.69 ± 1.64 (*p* = 0.311)
Respiratory Rate (breaths/min)	16.43 ± 0.13	16.30 ± 0.13 (*p* = 0.428)	16.13 ± 0.14 (*p* = 0.102)
Systolic Blood Pressure (mmHg)	116.76 ± 1.76	113.91 ± 2.30 (*p* = 0.357)	107.82 ± 2.34 ** (*p* = 0.005)
Diastolic Blood Pressure (mmHg)	76.13 ± 1.91	71.47 ± 1.89 (*p* = 0.115)	70.21 ± 2.34 * (*p* = 0.046)
Body Weight (kg)	58.53 ± 1.78	58.99 ± 1.70 (*p* = 0.838)	58.57 ± 1.27 (*p* = 0.983)
Body Height (cm)	155.39 ± 0.99	155.39 ± 1.00 (*p* = 1.000)	155.47 ± 1.58 (*p* = 0.960)
Body Mass Index (BMI) (kg/m^2^)	24.17 ± 0.61	24.40 ± 0.61 (*p* = 0.800)	24.35 ± 0.65 (*p* = 0.845)

D2, D4: The function soup containing “Anthaplex” at the doses of 2 g and 4 g per serving. ** *p*-value < 0.01 (compared vs. placebo); * *p*-value < 0.05 (compared vs. placebo).

**Table 5:** Effect of the functional soup containing “Anthaplex” at the doses of 2 g and 4 g per serving on the latency and amplitude of N100 and P300 waves of event-related potential at an 8-week consumption period (*n* = 23/group).

Location	Wave	Treatment Group	Baseline	8-Week
Fz	N100 Latency (ms)	Placebo	106.40 ± 2.47	106.00 ± 2.08
		D 2 g per day	109.00 ± 2.37 (*p* = 0.404)	98.86 ± 2.22 * (*p* = 0.016)
		D 4 g per day	106.39 ± 2.35 (*p* = 0.539)	103.86 ± 1.79 (*p* = 0.459)
	N100 Amplitude (μV)	Placebo	7.84 ± 0.93	7.36 ± 0.85
		D 2 g per day	7.97 ± 1.04 (*p* = 0.922)	10.16 ± 0.72 * (*p* = 0.022)
		D 4 g per day	5.71 ± 0.69 (*p* = 0.097)	10.47 ± 0.93 * (*p* = 0.011)
	P300 Latency (ms)	Placebo	345.00 ± 4.07	344.04 ± 3.68
		D 2 g per day	341.54 ± 3.70 (*p* = 0.485)	343.78 ± 3.22 (*p* = 0.955)
		D 4 g per day	350.08 ± 2.45 (*p* = 0.300)	344.91 ± 2.91 (*p* = 0.852)
	P300 Amplitude (μV)	Placebo	22.04 ± 1.75	23.83 ± 1.66
		D 2 g per day	20.99 ± 2.16 (*p* = 0.702)	22.28 ± 1.57 (*p* = 0.472)
		D 4 g per day	21.00 ± 1.78 (*p* = 0.702)	23.70 ± 1.23 (*p* = 0.953)
Cz	N100 Latency (ms)	Placebo	106.36 ± 2.53	102.47 ± 2.94
		D 2 g per day	110.77 ± 2.51 (*p* = 0.207)	104.34 ± 2.12 (*p* = 0.585)
		D 4 g per day	106.60 ± 2.23 (*p* = 0.943)	104.73 ± 2.04 (*p* = 0.509)
	N100 Amplitude (μV)	Placebo	7.84 ± 1.11	6.04 ± 0.91
		D 2 g per day	7.46 ± 0.88 (*p* = 0.897)	9.55 ± 0.99 ** (*p* = 0.001)
		D 4 g per day	6.10 ± 0.75 (*p* = 0.233)	8.25 ± 0.73 * (*p* = 0.014)
	P300 Latency (ms)	Placebo	341.60 ± 3.48	344.85 ± 3.40
		D 2 g per day	346.36 ± 3.52 (*p* = 0.331)	341.39 ± 3.83 (*p* = 0.486)
		D 4 g per day	349.71 ± 3.29 (*p* = 0.104)	347.77 ± 3.15 (*p* = 0.562)
	P300 Amplitude (μV)	Placebo	19.57 ± 1.98	21.25 ± 1.66
		D 2 g per day	19.95 ± 1.94 (*p* = 0.875)	20.14 ± 1.90 (*p* = 0.652)
		D 4 g per day	19.55 ± 1.63 (*p* = 0.774)	22.83 ± 1.53 (*p* = 0.510)

D2, D4: The function soup containing “Anthaplex” at the doses of 2 g and 4 g per serving. ** *p*-value < 0.01 (compared vs. placebo); * *p*-value < 0.05 (compared vs. placebo).

**Table 6:** Effect of the functional soup containing “Anthaplex” on working memory assessed by using event-related potential. (*n* = 23/group) * *p*-value < 0.05; compared to placebo group.

Cognitive Domains	Test Items	Treatment Group	Baseline	8-Week
Word Recognition	Time (ms)	Placebo	1497.31 ± 72.09	1300.95 ± 59.80
		D2 g per day	1331.22 ± 64.44 (*p* = 0.110)	1186.96 ± 49.87 (*p* = 0.132)
		D4 g per day	1453.52 ± 83.43 (*p* = 0.677)	1300.04 ± 47.14 (*p* = 0.555)
	%Accuracy	Placebo	83.96 ± 2.29	86.37 ± 2.33
		D2 g per day	84.63 ± 1.59 (*p* = 0.962)	87.24 ± 1.89 (*p* = 1.000)
		D4 g per day	83.01 ± 2.04 (*p* = 0.656)	85.30 ± 1.43 (*p* = 0.329)
Picture Recognition	Time (ms)	Placebo	1436.86 ± 50.65	1322.56 ± 48.01
		D2 g per day	1359.25 ± 68.57 (*p* = 0.204)	1291.03 ± 58.21 (*p* = 0.386)
		D4 g per day	1489.69 ± 67.97 (*p* = 0.496)	1308.66 ± 43.16 (*p* = 0.393)
	%Accuracy	Placebo	88.47 ± 1.84	85.68 ± 2.20
		D2 g per day	86.52 ± 1.84 (*p* = 0.501)	87.17 ± 1.36 (*p* = 0.920)
		D4 g per day	87.39 ± 1.82 (*p* = 0.727)	86.52 ± 1.42 (*p* = 0.893)
Simple Reaction	Time (ms)	Placebo	760.12 ± 32.36	727.65 ± 28.96
		D2 g per day	703.79 ± 22.26 (*p* = 0.135)	635.73 ± 18.94 * (*p* = 0.010)
		D4 g per day	730.33 ± 21.89 (*p* = 0.410)	695.93 ± 21.16 (*p* = 0.455)
Digit Vigilance	Time (ms)	Placebo	665.50 ± 14.05	655.04 ± 21.67
		D2 g per day	672.60 ± 9.72 (*p* = 0.648)	665.14 ± 9.79 (*p* = 0.538)
		D4 g per day	682.82 ± 8.46 (*p* = 0.267)	685.99 ± 7.69 (*p* = 0.468)
	%Accuracy	Placebo	91.24 ± 1.31	91.47 ± 1.44
		D2 g per day	92.75 ± 1.10 (*p* = 0.460)	93.97 ± 0.97 (*p* = 0.348)
		D4 g per day	90.91 ± 1.04 (*p* = 0.620)	93.75 ± 0.89 (*p* = 0.528)
Choice Reaction Time	Time (ms)	Placebo	884.09 ± 26.89	886.87 ± 32.03
		D2 g per day	862.69 ± 25.46 (*p* = 0.474)	864.63 ± 33.38 (*p* = 0.603)
		D4 g per day	885.38 ± 24.22 (*p* = 0.981)	869.68 ± 23.71(*p* = 0.688)
	%Accuracy	Placebo	97.21 ± 0.71	98.17 ± 0.46
		D2 g per day	97.65 ± 0.44 (*p* = 0.909)	97.50 ± 0.51 (*p* = 0.298)
		D4 g per day	97.69 ± 0.57 (*p* = 0.789)	97.43 ± 0.66 (*p* = 0.454)
Spatial Memory	Time (ms)	Placebo	1503.98 ± 68.44	1439.15 ± 77.30
		D2 g per day	1450.06 ± 60.10 (*p* = 0.633)	1452.06 ± 58.35 (*p* = 0.606)
		D4 g per day	1548.42 ± 70.67 (*p* = 0.683)	1433.48 ± 52.32 (*p* = 0.668)
	%Accuracy	Placebo	90.17 ± 2.11	85.43 ± 3.23
		D2 g per day	90.33 ± 2.46 (*p* = 0.617)	86.78 ± 2.70 (*p* = 0.903)
		D4 g per day	91.63 ± 1.69 (*p* = 0.765)	85.78 ± 2.87 (*p* = 0.722)
Numeric Working Memory	Time (ms)	Placebo	1200.45 ± 46.17	1131.49 ± 42.36
		D2 g per day	1179.57 ± 58.96 (*p* = 0.462)	1171.98 ± 48.10 (*p* = 0.470)
		D4 g per day	1207.86 ± 33.96 (*p* = 0.684)	1166.01 ± 23.54 (*p* = 0.538)
	%Accuracy	Placebo	93.04 ± 2.41	89.55 ± 2.82
		D2 g per day	93.91 ± 1.71 (*p* = 0.477)	96.07 ± 1.15 * (*p* = 0.015)
		D4 g per day	92.17 ± 2.07 (*p* = 0.311)	94.92 ± 0.87 * (*p* = 0.046)

D2, D4: The function soup containing “Anthaplex” at the doses of 2 g and 4 g per serving. (*n* = 23/group) * *p*-value < 0.05 (compared vs. placebo).

**Table 7:** Severity score of eye dryness via the modified criteria of Messmer (2015) by physician. Data were expressed as mean ± SEM. (*n* = 23/group).

Treatment	Severity Score at Baseline	Severity Score after an 8 Week-Consumption Period
Placebo	4.47 ± 0.14	4.42 ± 0.11
D2 g per day	4.72 ± 0.17	4.22 ± 0.11 #
D4 g per day	4.47 ± 0.11	4.23 ± 0.10

D2, D4: The function soup containing “Anthaplex” at the doses of 2, and 4 g per serving. # *p*-value < 0.05 (compared vs. baseline data).

**Table 8:** The effect of the functional soup containing various doses of “Anthaplex” on acetylcholinesterase (AChE), monoamine oxidase, monoamine oxidase type A and type B (MAO-A, MAO-B), GABA transaminase (GABA-T) and brain-derived neurotropic factor (BDNF). Data were expressed as mean + SEM). (*n* = 23/group) * *p*-value < 0.05 compared to placebo group.

Parameters	Times	Placebo	D2 g per Day	D4 g per Day
AChE activity (nmol/mg protein)	Baseline	6.43 ± 0.50	5.54 ± 0.40 (*p* = 0.249)	5.97 ± 0.47 (*p* = 0.511)
	8-week	6.35 ± 0.34	5.33 ± 0.27 * (*p* = 0.016)	6.07 ± 0.23 (*p* = 0.525)
MAO activity (umol/mg protein)	Baseline	4.03 ± 0.34	3.66 ± 0.23 (*p* = 0.849)	3.44 ± 0.23 (*p* = 0.166)
	8-week	2.90 ± 0.16	2.89 ± 0.16 (*p* = 0.988)	2.73 ± 0.09 (*p* = 0.782)
MAO-A activity (umol/mg protein)	Baseline	1.85 ± 0.36	1.48 ± 0.22 (*p* = 0.988)	1.26 ± 0.22 (*p* = 0.328)
	8-week	1.27 ± 0.20	1.15 ± 0.17 (*p* = 0.620)	0.98 ± 0.12 (*p* = 0.465)
MAO-B activity (umol/mg protein)	Baseline	1.22 ± 0.19	0.99 ± 0.11 (*p* = 0.919)	0.93 ± 0.12 (*p* = 0.474)
	8-week	0.89 ± 0.10	0.82 ± 0.10 (*p* = 0.605)	0.82 ± 0.08 (*p* = 0.612)
GABA-T (nmol/mg protein)	Baseline	0.22 ± 0.02	0.19 ± 0.01 (*p* = 0.988)	0.18 ± 0.01 (*p* = 0.165)
	8-week	0.18 ± 0.01	0.18 ± 0.01 (*p* = 0.838)	0.16 ± 0.00 (*p* = 0.726)
BDNF (pg/mL)	Baseline	113.23 ± 3.82	118.74 ± 4.36 (*p* = 0.411)	113.07 ± 5.48 (*p* = 0.979)
	8-week	117.21 ± 4.18	146.20 ± 12.57 * (*p* = 0.033)	143 ± 9.25 * (*p* = 0.043)

D2, D4: The function soup containing “Anthaplex” at the doses of 2, and 4 g per serving.

**Table 9:** The effect of the functional soup containing various doses of “Anthaplex” on feces pH, amount of lactic-acid-producing bacteria, and the amount of both *Lactobacillus* spp. and *Bifidobacterium* spp. Data were presented as mean + SEM). (*n* = 23/group) *, ** *p*-value < 0.05, 0.01 respectively; compared to placebo group.

Parameters	Treatment Duration	Placebo	D2 g per Day	D4 g per Day
pH	Baseline	7.43 ± 0.07	7.19 ± 0.07	7.35 ± 0.08
	8-week	6.83 ± 0.12	6.74 ± 0.08 * (*p* = 0.02)	6.70 ± 0.07 * (*p* = 0.026)
Amount of lactic acid producing bacteria (Log of CFU/mL)	Baseline	8.13 ± 0.23	7.88 ± 0.17 (*p* = 0.734)	8.00 ± 0.17 (*p* = 0.865)
	8-week	7.76 ± 0.17	7.56 ± 0.15 (*p* = 0.147)	7.55 ± 0.14 (*p* = 0.482)
Amount of *Lactobacillus* spp. (Log of CFU/mL)	Baseline	6.64 ± 0.29	6.79 ± 0.08 (*p* = 0.918)	6.71 ± 0.24 (*p* = 0.564)
	8-week	6.64 ± 0.30	6.72 ± 0.18 (*p* = 0.825)	6.62 ± 0.32 (*p* = 0.363)
Amount of *Bifidobacterium* spp. (Log of CFU/mL)	Baseline	6.84 ± 0.56	6.56 ± 0.05 (*p* = 0.934)	6.68 ± 0.25 (*p* = 0.998)
	8-week	6.61 ± 0.04	7.76 ± 0.43 ** (*p* = 0.006)	6.69 ± 0.20 (*p* = 0.417)

D2, D4: The function soup containing “Anthaplex” at the doses of 2, and 4 g per serving.

**Table 10:** The effect of the functional soup containing various doses of “Anthaplex” on short-chain fatty acids (SCFA) consisting of acetate, propionate, and butyrate. Data were presented as mean ± SEM. (*n* = 23/group) * *p*-value < 0.05; compared to placebo group.

Parameters	Time Window of Treatment	Placebo	D2 g/Day	D4 g/Day
Acetate (mM)	Baseline	4.70 ± 0.88	6.76 ± 1.37 (*p* = 0.351)	6.74 ± 1.49 (*p* = 0.313)
	8-week	6.79 ± 1.01	7.55 ± 1.13 (*p* = 0.598)	8.57± 0.83 (*p* = 0.210)
Propionate (mM)	Baseline	2.27 ± 0.52	3.64 ± 0.83 (*p* = 0.235)	3.70 ± 0.70 (*p* = 0.181)
	8-week	3.09 ± 0.44	3.64± 0.67 (*p* = 0.487)	4.85 ±0.53 * (*p* = 0.032)
Butyrate (mM)	Baseline	1.64 ± 0.30	3.10 ± 0.93 (*p* = 0.206)	3.36 ± 0.61 (*p* = 0.835)
	8-week	2.72 ± 0.52	3.49 ± 0.58 (*p* = 0.311)	3.33 ± 0.43 (*p* = 0.405)

D2, D4: The function soup containing “Anthaplex” at the doses of 2 g and 4 g/serving.

**Table 11:** The effect of the functional soup containing various doses of “Anthaplex” on histone acetyltransferase (HAT), histone deacetylase (HDAC), and DNA methyltransferase (DNMT). Data were presented as mean + SEM. (*n* = 23/group) * *p*-value < 0.05; compared to placebo group.

Parameters	Times	Placebo	D2 g/Day	D4 g/Day
Histone Acetyltransferase (HAT)	Baseline	1.03 ± 0.048	1.09 ± 0.77 (*p* = 0.415)	1.03 ± 0.039 (*p* = 0.967)
	8-week	0.96 ± 0.042	1.06± 0.072 (*p* = 0.192)	1.06 ± 0.036 (*p* = 0.922)
Histone Deacetylase (HDAC)	Baseline	7.41 ± 0.72	6.16 ± 0.51 (*p* = 0.171)	7.27 ± 0.62 (*p* = 0.876)
	8-week	7.48 ± 0.59	5.77± 0.48 * (*p* = 0.040)	6.14 ± 0.57 (*p* = 0.104)
DNA Methyltransferase (DNMT)	Baseline	0.35 ± 0.05	0.35 ± 0.05 (*p* = 0.952)	0.32 ± 0.05 (*p* = 0.745)
	8-week	0.29 ± 0.04	0.32± 0.05 (*p* = 0.734)	0.26± 0.04 (*p* = 0.732)

## Data Availability

I confirm that data are available and will be provided on request because all data are in the process of petty patent registration during this period.
